# Cytogenetic Profile of *de novo* Acute Myeloid Leukemia Patients in Malaysia

**Published:** 2013-03

**Authors:** Chin Yuet Meng, Puteri J. Noor, Azli Ismail, Mohd Fadly Md Ahid, Zubaidah Zakaria

**Affiliations:** *Hematology Unit, Cancer Research Centre, Institute for Medical Research, Kuala Lumpur, Malaysia*

**Keywords:** acute myeloid leukemia (AML), chromosome abnormalities, age

## Abstract

Acute myeloid leukemia (AML) is a heterogeneous disease in terms of cytogenetics and molecular genetics. AML is the most common acute leukemia in adults and its incidence increases with age. Diagnostic cytogenetics is an important prognostic indicator for predicting outcome of AML. We examined the karyotypic patterns of 480 patients with *de novo* AML seen at government hospitals throughout the country and evaluated the association of chromosome aberrations with the age of patient. Chromosome abnormalities were detected in 146 (30.4%) patients. The most common cytogenetic abnormality was balanced translocation t (8; 21), followed by trisomy 8 (as sole abnormality) and t (15; 17). The age of our Malaysian patients at diagnosis ranged from four months to 81 years, with a median age of 39 years. The normal karyotype was found mainly in patients aged 15-30 years. About 75% of patients with t (8; 21) were below 40 years of age, and the complex karyotype was found with the highest frequently (34.3%) in elderly patients (age above 60 years). More than half of the patients with complex karyotype were above 50 years of age. The deletion 5q was detected only in patients aged above 50 years. Different cytogenetic abnormalities in AML show different frequencies with increasing age. Probably different genetic mechanisms are involved in the pathogenesis of AML and these mechanisms might occur at different frequencies over lifetime.

## INTRODUCTION

Acute myeloid leukemia (AML) is cancer of the myeloid blood cells, and is characterized by the rapid growth of abnormal white blood cells in the bone marrow, thus interfering with the production of normal blood cells. The incidence of AML in the white population (3.8 per 100,000 person) is higher than that of the Asian population (3.2 per 100,000 person) (1). AML is the most common acute leukemia in adult and also it is more common in males than females. The incidence increases with age, with the majority of patients older than 60 years. In general, the five-year survival rate is about 20-25%. However, in elderly patients the survival is even worse. The disease is heterogeneous in terms of morphology, immunophenotype, cytogenetics, molecular genetics and clinical features. The classification of AML is based on the World Health Organization (WHO) classification of the myeloid neoplasms and acute leukemia (2). Using the WHO criteria, the diagnosis of AML is established by the presence of 20% or more of leukemic myeloblasts in the peripheral blood (PB) and / or bone marrow (BM). However, in a subgroup of AML, the presence of recurrent genetic abnormalities alone such as translocation between chromosomes 8 and 21 t (8; 21) in AML, inversion of chromosome 16 [inv (16)] or t (16; 16) in AML, and t (15; 17) in acute promyelocytic leukemia (APL), is sufficient for the diagnosis of AML regardless of the blast percentage in the PB or BM. Three new cytogenetically defined entities are added to the diagnosis and classification of AML in the revised WHO classification of myeloid neoplasms and acute leukemia: AML with t(6;9), AML with inv (3) or t (3; 3), and AML (megakaryoblastic) with t (1; 22) (2). Risk factors for developing AML include a history of preleukemic blood disorders such as myelodysplastic syndrome, past treatment with chemotherapy or radiation therapy, exposure to ionizing radiation or chemical such as benzene, and genetics (3). Congenital conditions such as Down syndrome is associated with a 10- to 20-fold increased risk of leukemia (4).

Diagnostic karyotype is one of the most powerful prognostic indicators for predicting outcome of AML. Certain chromosome abnormalities are associated with good outcomes while other chromosome abnormalities are associated with a poor prognosis and a high risk of relapse. The three risk groups and the chromosome abnormalities associated with them are: Favourable risk group: t (8; 21), t (15; 17), inv (16) or t (16;16); Intermediate risk group: Normal karyotype, t (9; 11), -Y (loss of the Y chromosome), +8 (trisomy of chromosome 8), +11, +13, +21, del (7q) [deletion of the long arm of chromosome 7], del (9q), and del (20q); and Unfavourable risk group: Complex karyotype, inv (3) or t (3; 3), t (6; 9), t (6; 11), t (11; 19), del (5q), -5 (Monosomy of chromosome 5), -7 (5-7). In the favourable risk group, the presence of additional chromosomal changes had no significant impact on APL patients with t (15; 17) when treated with all-trans retinoic acid (ATRA) and anthracycline–based protocols. Additional chromosomal aberrations also did not have any adverse effect on t (8; 21) AML. However, a even better prognosis was observed in AML patients with inv(16) having additional chromosomal changes (particularly trisomy 22) (8). Within each cytogenetic risk group, the prognosis decreases with increasing age. With increasing age, there is an increase in the proportion of patients with unfavourable risk cytogenetics and a decrease in favourable risk cytogenetics (9). In this study we evaluated the common chromosome aberrations found in AML patients at presentation of the disease, and the association of different chromosome abnormalities with age of the AML patients.

## MATERIALS AND METHODS

### Patients

Cytogenetic studies were performed as a routine diagnostic test for all patients with hematological malignancies at presentation of disease as well as in all follow up cases by our Cytogenetics Laboratory, Hematology Unit, Institute for Medical Research (IMR), Kuala Lumpur. The samples received by our Cytogenetics Laboratory came from mainly the Government Hospitals throughout the country. Bone marrow samples from 480 AML patients at presentation of disease sent to the IMR for routine cytogenetic studies from the year 2007 to 2012 were included in this study. The AML patients include both the pediatric (≤14 years of age) and adult cases (age above 14 years). Secondary AML after a myelodysplastic syndrome, therapy related AML, and patients with Down Syndrome who developed AML were excluded from this study. The diagnosis of AML was according to the WHO classification with the incorporation of morphology, cytochemistry, immunophenotype, cytogenetics and clinical data.

### Cytogenetic studies

Conventional cytogenetic analysis (CCA) was performed according to standard techniques (10). The bone marrow cells were cultured overnight without the addition of any stimulating agent to make the cells undergo mitosis. The chromosomes were G-banded and karyotyping was done according to the International System for Human Cytogenetic Nomenclature (ISCN) (11).

### Statistics

Cytogenetic findings were classified into five subgroups: Normal karyotype, balanced translocations, unbalanced but non-complex aberrations, complex karyotype, and other aberrations (12). The balanced translocations comprised of t (8; 21), t (15; 17), inv (16), 11q23 rearrangements (aberrations in band 23 of the long arm of chromosome 11) and other rare balanced translocations. The unbalanced but non complex group include the following as the sole abnormality: Single trisomies, single monosomies of chromosome 7 or del (7q), single monosomies of chromosome 5 or del (5q), and other single monosomies. Three or more unrelated chromosome abnormalities, none of which were included in the ‘AML with recurrent genetic abnormalities’ subgroup was defined as complex karyotype (2). The ‘other aberrations’ group comprised of single structural aberrations or a combination of different cytogenetic abnormalities.

Patients were divided into six age groups, Age group 1: ≤14 years, age group 2: 15–30 years, age group 3: 31-40 years, age group 4: 41-50 years, age group 5: 51-60 years, and age group 6: ≥61 years. Age group 1 are the pediatric AML patients (≤14 years). Patients in group 6 are the elderly AML patients. AML patients above 60 years were defined as elderly according to The National Comprehensive Cancer Network (NCCN) guidelines (9).

The percentage of cytogenetic subtypes in the different age groups were calculated. Differences in the cytogenetic subtypes among the different age groups were analysed by chi square. *p*- values less than 0.05 were considered as statistically significant.

## RESULTS

### Cytogenetic findings in AML patients

Out of the 480 AML patients at diagnosis, 245 (51.0%) patients were male and 235 (49.0%) patients were female. The age of the patients ranged from four months to 81 years, with a median age of 39 years. Chromosome abnormalities were detected in 146 (30.4%) patients while 334 (69.6%) patients had a normal karyotype. The frequency of the chromosome abnormalities found were as follows: Balanced translocations, 56 (11.7%) patients; unbalanced aberrations, 32 (6.6%) patients; complex karyotype, 35 (7.3%) patients; and other aberrations, 23 (4.8%) patients (Table 1). In the balanced translocation group of 56 patients, 36 (7.5%) patients had t (8; 21), 11 (2.3%) patients had translocation t (15; 17), and 9 (1.9%) patients had other type of balanced translocation. The unbalanced but non complex group of 32 patients comprised of 20 (4.2%) patients with single trisomies, six (1.2%) patients with either -7 or del (7q), four (0.8%) patients with del (5q), and two (0.4%) patients with other single monosomies. The single trisomies group comprised of fourteen trisomy 8 (3.0%), two trisomy 10 (0.4%), two trisomy 11 (0.4%), one trisomy 21 (0.2%), and one trisomy 22 (0.2%).

**Table 1:** Cytogenetic findings in acute myeloid leukemia patients

*Cytogenetic subtype*	*n*	*% of all patients*

**Normal karyotype**	**334**	**69.6**

t (8; 21)	36	7.5%
t (15; 17)	11	2.3%
Other balanced translocations	9	1.9%
**Balanced translocations**	**56**	**11.7%**
Trisomy 8	14	3.0%
Other trisomies	6	1.2%
-7/del (7q)	6	1.2%
-5/del (5q)	4	0.8%
Other monosomies	2	0.4%
**Unbalanced aberrations**	**32**	**6.6%**

**Complex karyotype**	**35**	**7.3%**

**Other aberrations**	**23**	**4.8%**

***Total***	***480***	***100.0***

n: number of patients.

### Frequency of chromosome aberrations in cytogenetically abnormal AML

The frequency of chromosome aberrations in the 146 cytogenetically abnormal AML were as follows (Table 2): t (8; 21), 24.6%; t (15; 17), 7.5%; other balanced translocations, 6.2%; trisomy 8, 9.6%; other trisomies, 4.1%; -7 or del (7q), 4.1%; -5 or del (5q), 2.7%; other monosomies, 1.4%; complex karyotype, 24.0%; and other aberrations, 15.8%.

**Table 2:** Frequency of chromosome aberrations in cytogenetically abnormal acute myeloid leukemia

*Types of chromosome aberrations*	*n*	*% of Patients*

t (8; 21)	36	24.6%
t (15; 17)	11	7.5%
Other balanced translocations	9	6.2%
**Balanced translocations**	**56**	**38.3%**

Trisomy 8	14	9.6%
Other trisomies	6	4.1%
-7/del (7q)	6	4.1%
-5/del (5q)	4	2.7%
Other monosomies	2	1.4%
**Unbalanced aberrations**	**32**	**21.9%**

**Complex karyotype**	**35**	**24.0%**

**Other aberrations**	**23**	**15.8%**

***TOTAL***	***146***	***100%***

n: number of patients.

### Frequency of cytogenetic aberrations in the six age groups

The number of patients diagnosed in each of the six age groups were as follows: ≤14 years, 61 (12.7%) patients; 15–30 years, 121 (25.2%) patients; 31–40 years, 75 (15.6%) patients; 41–50 years, 82 (17.1%) patients; 51–60 years, 70 (14.6%) patients; and 61 years and above, 71 (14.8%) patients. The proportion of the six age groups within the different cytogenetic subgroups were analysed (Table 3). Age group 2 (15-30 years) had the highest frequency of normal karyotype (25.2%) compared to the other age groups (*p*<0.0001). The translocation t (8; 21) was found mainly in the younger age group. About 75% of patients with translocation t (8; 21) were below the age of 40 years. The deletion 5q was detected only in patients aged above 50 years, while -7/del (7q) was found mainly in patients aged 15–30 years (66.6%). Complex karyotype was found with the highest frequency (34.3%) in age group 6 (above 60 years) when compared to the other age groups (*p*<0.05). About 57.2% of patients with complex karyotype were aged above 50 years. The other aberration group which comprised of 23 patients were excluded from further evaluation (12).

**Table 3:** Proportions of the different cytogenetic subtypes in each age group

*Cytogenetic subgroups*	*n*	*Age Group 1 (≤14 yrs)*	*Age Group 2 (15-30 yrs)*	*Age Group 3 (31-40 yrs)*	*Age Group 4 (41-50yrs)*	*Age Group 5 (51-60yrs)*	*Age Group 6 (61yrs & above)*	*All Age Groups*

**Normal karyotype**	**334**	**35 (10.5%)**	**86 (25.7%)**	**59 (17.7%)**	**64 (19.2%)**	**46 (13.8%)**	**44 (13.1%)**	**100%**

T (8;21)	36	12 (33.4%)	9 (25.0%)	6 (16.7%)	2 (5.5%)	5 (13.9%)	2 (5.5%)	100%
t(15;17)	11	2 (18.2%)	3 (27.2%)	2 (18.2%)	2 (18.2%)	1 (9.1%)	1 (9.1%)	100%
Other Balanced translocations	9	2 (22.2%)	2 (22.2%)	1 (11.1%)	1 (11.1%)	2 (22.2%)	1 (11.2%)	100%
**Balanced translocations (BT)**	**56**	**16 (28.6%)**	**14 (25.0%)**	**9 (16.1%)**	**5 (8.9%)**	**8 (14.3%)**	**4 (7.1%)**	**100%**

Trisomies	20	2 (10.0%)	6 (30.0%)	1 (5.0%)	2 (10.0%)	3 (15.0%)	6 (30.0%)	100%
-7/del(7q)	6	-	4 (66.6%)	-	1 (16.7%)	1 (16.7%)	-	100%
del(5q)	4	-	-	-	-	3 (75.0%)	1 (25.0%)	100%
Other monosomies	2	-	1 (50.0%)	-	-	-	1 (50%)	100%
**Unbalanced aberrations**	**32**	**2 (6.3%)**	**11 (34.4%)**	**1 (3.1%)**	**3 (9.4%)**	**7 (21.8%)**	**8 (25.0%)**	**100%**

**Complex karyotype**	**35**	**5 (14.3%)**	**4 (11.4%)**	**1 (2.8%)**	**5 (14.3%)**	**8 (22.9%)**	**12 (34.3%)**	**100%**

**Other aberrations**	**23**	**3 (13.1%)**	**6 (26.1%)**	**5 (21.7%)**	**5 (21.7%)**	**1 (4.3%)**	**3 (13.1%)**	**100%**

***TOTAL***	***480***	***61 (12.7%)***	***121 (25.2%)***	***75 (15.6%)***	***82 (17.1%)***	***70 (14.6%)***	***71 (14.8%)***	***100%***

n: number of patients; yrs: years.

## DISCUSSION

The incidence of all leukemia in the white population (13.5 per 100,000 person) is higher than that of the Asian population (7.5 per 100,000 person) (1). In the United Kingdom the incidence of AML is 3.0 per 100,000 people (13). About 25% of adult with leukemia is AML in the Western population. In Malaysia leukemia is the seventh most common cancer, with an incidence of only 2.9 per 100,000 population for all leukemia (14). The low incidence of leukemia in the Malaysian population could be due to geographical, environmental and ethnic differences. The National Census carried out in the year 2010 showed the Malaysian population consist mainly of the ethnic groups Bumiputera (67.4%), Chinese (24.6%), Indians (7.3%) and others (0.7%) (15). The Bumiputera is also a heterogeneous group, with the Malays being the predominant ethnic group.

The median age of our Malaysian AML patients at presentation of the disease was younger, 39 years compared to 65-70 years in the Western population (16). This could be due to ethnic, geographic, and demographic differences. The median age of our general population was younger (26.2 years) when compared to the Western population (40 years) in the year 2010 (15, 17). The incidence of AML is expected to increase as the population ages. The National Census 2010 showed that the Malaysian population is aging, and we may expect to see an increase in the incidence of AML in the future.

Chromosome abnormalities were detected in 30.4% of our AML patients. Studies from other geographical regions have reported higher chromosome abnormalities in AML, more than 50% (18, 19). The most common cytogenetic abnormality was t (8; 21) [7.5%], followed by trisomy 8 (3.0%), and t (15; 17) [2.3%] (Table 1). Trisomy 8 is the most common numerical aberration in AML and is present as a sole abnormality in 6% of newly diagnosed cytogenetically abnormal AML (20). In our study, trisomy 8 is also the most common sole numerical abnormality with a frequency of 9.6% in *de novo* cytogenetically abnormal AML (Table 2). Trisomy 8 is considered as an intermediate cytogenetic-risk alteration and its pathogenetic role is still unclear. The favourable prognostic impact of t (8; 21), inv (16), and t (15; 17) in AML is not modified by the presence of an additional trisomy 8. In newly diagnosed AML, -7/del (7q) and -5/del (5q) are found as sole chromosome abnormality in 4-5% and 6-9% respectively, of all chromosomal abnormalities (21). In our *de novo* AML, about 4.1% had -7/del (7q) as the sole chromosomal abnormality, and this was also similar to that of reported cases. Chromosome aberrations involving -7/del (7q) and -5/del (5q) are frequently observed in patients exposed to alkylating agents and carcinogens. It is well known that balanced translocations tend to be found in younger AML patients, while elderly patients usually have unbalanced aberrations such as complex karyotype. This was also found in our study, 75% of patients with the balanced translocation t (8; 21) were detected in patients below 40 years of age. Patients with complex karyotype were found with the highest frequency (34.3%) in age group 6 (the elderly patients). Complex karyotype is found in about 10-12% of AML patients and is considered a high-cytogenetic risk aberration with a poor prognosis (21). About 7.3% of AML patients in our study had a complex karyotype. The deletion 5q was only found in patients above 50 years of age. We did not detect any monosomy 5 as the sole cytogenetic aberration in our study. Bacher *et al.* 2005(12) reported a 91.3 fold increase in the incidence of del(5q) in their elderly AML patients when compared to that of patients in age group 31–40 years. Probably the mechanisms leading to balanced translocations and unbalanced aberrations are different from each other, and may be due to age-associated factors.

About 69.6% of AML patients in our study had a normal karyotype by CCA. Studies from other countries have reported a normal karyotype in AML with a frequency of about 35–45% (8, 22). The lower frequency of chromosomal aberrations and the higher frequency of normal karyotype in AML patients in our Malaysian population compared to that of other studies could be due to the limitations of CCA, ethnic, geographical and demographic differences. Missed chromosome aberrations in AML with a normal karyotype could be due to three major factors (23). First, numerical aberrations such as trisomy 8 and trisomy11 have been reported in the interphase cells of AML with normal karyotypes, probably due to the inability of the abnormal clone with aneuploidy to proliferate *in vitro* (24). Second, the quality of the chromosome morphology and the G-banding resolution may also result in aberrations not detected by CCA. For example, the t (11; 19) which is a recurrent abnormality is detectable only in high quality chromosome morphology. *TEL* deletion on chromosome 12p had been detected by FISH (fluorescence *in situ* hybridization) studies in AML with normal karyotype (25). Third, a normal karyotype is observed in some AML due to cryptic rearrangements. Cryptic rearrangements have been observed in t (8; 21), inv (16) and t (15; 17) with incidences of 0 to 4% (26, 27). 11q23 rearrangements are present in 4-10% of AML patients (21). The most common 11q23 rearrangements are t (9; 11), t (6; 11), t (10; 11) and t (11; 19). We did not detect any 11q23 rearrangements in our study so far and inv (16) was rarely seen. This could probably be due to the short chromosomes frequently seen in our preparation. The advantage of CCA is that it has the intrinsic ability to detect any structural or numerical aberration, novel and uncharacterized abnormalities. However, cytogenetics findings in AML still remain as a corner stone in predicting prognosis.

The cytogenetically normal karyotype in AML is a heterogeneous group in terms of response to treatment, achievement of complete remission, and relapse rate. It is classified as intermediate cytogenetic risk. Clinical outcome of AML patients with normal cytogenetics has been shown to be affected by molecular genetics alterations (28, 29). Molecular mutations were proven for the first time by Bacher *at al*. (30) to be prognostically relevant in patients with aberrant karyotypes in the intermediate cytogenetic risk group. Molecular studies must be integrated with cytogenetic studies for risk stratification at diagnosis to improve therapeutic strategies.

In conclusion, the median age at diagnosis of AML in our Malaysian patients was 39 years. The three most common chromosome abnormalities detected in AML were t(8;21), trisomy 8, and t(15;17). Age related cytogenetic subtypes such as balanced translocation t(8;21) was found more frequently in younger AML patients, while complex karyotype was usually found in elderly patients. Probably different genetic mechanisms are involved in the pathogenesis of AML and these mechanisms might occur at different frequencies as age increases.
